# Genetically modified DP915635 maize is agronomically and compositionally comparable to non-genetically modified maize

**DOI:** 10.1080/21645698.2023.2208997

**Published:** 2023-05-04

**Authors:** Jennifer A. Anderson, James Mickelson, Brandon J. Fast, Nathan Smith, Robert C. Pauli, Carl Walker

**Affiliations:** Corteva Agriscience™, Johnston, IA, USA

**Keywords:** agronomics, composition, genetically modified, safety assessment, substantial equivalence, *Zea mays* L.

## Abstract

DP915635 maize was genetically modified (GM) to express the IPD079Ea protein for corn rootworm (Diabrotica spp.) control. DP915635 maize also expresses the phosphinothricin acetyltransferase (PAT) protein for tolerance to glufosinate herbicide and the phosphomannose isomerase (PMI) protein that was used as a selectable marker. A field study was conducted at ten sites in the United States and Canada during the 2019 growing season. Of the 11 agronomic endpoints that were evaluated, two of them (early stand count and days to flowering) were statistically significant compared with the control maize based on unadjusted p-values; however, these differences were not significant after FDR-adjustment of p-values. Composition analytes from DP915635 maize grain and forage (proximates, fiber, minerals, amino acids, fatty acids, vitamins, anti-nutrients, and secondary metabolites) were compared to non-GM near-isoline control maize (control maize) and non-GM commercial maize (reference maize). Statistically significant differences were observed for 7 of the 79 compositional analytes (16:1 palmitoleic acid, 18:0 stearic acid, 18:1 oleic acid, 18:2 linoleic acid, 24:0 lignoceric acid, methionine, and α-tocopherol); however, these differences were not significant after FDR-adjustment. Additionally, all of the values for composition analytes fell within the range of natural variation established from the in-study reference range, literature range, and/or tolerance interval. These results demonstrate that DP915635 is agronomically and compositionally comparable to non-GM maize represented by non-GM near-isoline control maize and non-GM commercial maize.

## Introduction

Western corn rootworm (WCR; *Diabrotica virgifera virgifera*) is a detrimental insect pest of maize in the United States and Canada.^1^ Potential annual damage from this pest has been estimated at over 1 billion dollars in the United States and 450 million euros in Europe.^2–5^ Annual crop rotations and insecticides have been effective WCR control methods, and starting in 2003 several varieties of genetically modified maize expressing crystalline (cry) proteins produced by *Bacillus thuringiensis* (*Bt*) have also been used to control WCR.^1,6^ However, WCR has adapted to each of these management practices and insecticide resistant, Bt-resistant, and crop rotation-resistant phenotypes have emerged.^7–12^ Therefore, novel management practices and new modes of action against WCR are needed to help control this damaging insect pest.

Event DP-915635-4 (DP915635) maize expresses the IPD079Ea protein, which is encoded by the *ipd079Ea* gene from *Ophioglossum pendulum* and is an insecticidal protein with activity against corn rootworm (CRW) pests. DP915635 maize also expresses the *mo-pat* and *pmi* genes, which encode the phosphinothricin acetyltransferase (PAT) and phosphomannose isomerase (PMI) proteins, respectively.^13^ The PAT protein confers tolerance to the herbicidal active ingredient glufosinate ammonium at current labeled rates and the PMI protein was used as a selectable marker during the development of DP915635 maize.

Event DP915635 represents one of the first genetically modified maize events to contain an insecticidal protein derived from a fern. *Ophioglossum pendulum*, known as the Old World adder’s-tongue fern, is found in the United States in the state of Florida and is native to India, Australia, parts of Africa, and Southeast Asia.^14,15^ Similar to the mode of action of 3-domain Cry toxins from *Bt*, the IPD079Ea protein is a pore-forming protein which localizes in the target insect midgut. The IPD079Ea protein contains a Membrane Attack Complex/Perforin and Cholesterol-Dependent Cytolysin domain (MACPF/CDC), which are widespread across bacteria and eukaryotes.^16^ Following the ingestion of DP915635 maize tissue, the IPD079Ea protein binds to receptors present in the midgut epithelial cells of CRW, and the subsequent disruption of the midgut epithelial cells caused by pore-formation results in insect death.

To satisfy the regulatory requirements for GM crop cultivation, a multi-location field trial was conducted to compare the agronomic characteristics of DP915635 maize with those of non-GM near-isoline control maize. The composition of grain and forage from DP915635 maize was also compared with that of grain and forage from non-GM near-isoline control maize to fulfill GM crop regulatory requirements. The results of the agronomic and composition evaluations are published to add to the weight of evidence that DP915635 maize is as safe and nutritious as non-GM maize.

## Materials and Methods

### Field Study

The field study was planted during the 2019 growing season at ten sites in the United States and Canada (Iowa, Indiana, Nebraska, Pennsylvania, two sites in Illinois, two sites in Texas, and two sites in Ontario), which were selected to represent North American growing regions for commercial maize. Each field site employed a randomized complete block design, and each block included DP915635 maize, control maize, and four reference maize hybrids. The control maize (non-GM near-isoline control) had the same genetic background as DP915635 maize (PH1KTF/PHR03) but did not contain the genetic modification and was used as a comparator to identify statistical differences. A total of 20 non-GM commercial maize hybrids (5513, P0506, 35A52, P0604, P0760, 5883, P0993, 5939, 5828, P1151, P1197, 6158, P0928, P1105, P1345, P1319, P1395, P1422, 33Y74, and 6575) were utilized for reference hybrids in the study. The four reference maize hybrids that were planted at each field site were selected from these 20 reference hybrids based on the maturity zone of each individual field site, as well as the Comparative Relative Maturity (CRM) of each hybrid. The reference maize hybrids represent a range of non-GM hybrids that are planted commercially and were used to establish ranges of the natural variation that occurs in non-GM maize.

All seeds were analyzed by event-specific polymerase chain reaction to confirm the presence of the event in the DP915635 maize and the absence of the event in the control maize. Plots consisted of six rows measuring 6.1 m in length and 0.76 m in width, with few exceptions. Each row was planted with 30 seeds. Blocks were separated by an alley of at least 0.9 m, and each plot was bordered on both sides by one row of maize. Plots were maintained following standard irrigation, fertilization, and herbicide and pesticide practices, and all maintenance products were uniformly applied to the entire trial as needed at each field site. Open-pollinated plants were used for agronomic assessments, whereas self-pollinated plants were used for composition sample collection.

### Agronomic assessment

Agronomic data were collected at each of the ten field sites of the study for 11 characteristics (early stand count, days to flowering, pollen viability, plant height, days to maturity, lodging, final stand count, dropped ears, yield, harvest grain moisture, and 100-kernel weight), as described previously^17^ and as summarized in Table 1 and Supplemental Information Table S1.

**Table 1.** Description of agronomic characteristics evaluated.

Characteristic Measured	Evaluation Timing^a^	Description (units of measure)
Early Stand Count	V2-V4	Number of plants emerged per plot (count/m^2^)
Days to Flowering	Approximately 50% pollen shed	Number of days from planting until approximately 50% of the plants have tassels shedding pollen (days)
Pollen Viability ^b^	During pollen shed	Pollen Shape: pollen grains with collapsed walls at 0, 30, 60, and 120 min (% of pollen with collapsed walls)
		Pollen Color: pollen grains with intense yellow color at 0, 30, 60, and 120 min (% of pollen yellow in color)
Plant Height	R4	Height from soil surface to the collar of flag leaf (cm)
Days to Maturity	R6	Number of days for the majority of the plants to first reach physiological maturity (days)
Lodging	R6	Combined score of stalk lodging (the number of plants in each plot with stalks broken below the primary ear) and root lodging (the number of plants in each plot with stalks leaning approximately 45 degrees or more) values (percentage)
Final Stand Count	R6	Number of plants remaining per plot (count/m^2^)
Dropped Ears	R6	Number of ears lying on the ground within each plot (count)
Yield	R6	Harvest weight per area adjusted to 15.5% moisture content (bushels per acre)
Harvest Grain Moisture	R6	Grain moisture content (percentage)
100-Kernel Weight	R6	Weight of 100 kernels (grams)

^a^Abendroth et al. ^37^ describes maize growth stages.

^b^Pollen viability has been correlated to pollen shape and color ^28^.

### Forage and Grain Sample Collection and Processing

Eight of the ten field sites in the study were selected for nutrient composition analysis (Iowa, Indiana, Nebraska, Pennsylvania, Texas, Ontario, and two sites in Illinois). Plants that were self-pollinated and were representative of the other plants within each plot were sampled for forage or grain. One forage sample, which consisted of all above-ground parts of three plants pooled together, was collected from each plot at the R4 growth stage, as described previously.^17^ One grain sample, which consisted of the grain from five husked and shelled ears pooled together, was collected from each plot at the R6 growth stage.^17^ Both forage and grain samples were placed in chilled storage (using wet ice, artificial ice, or dry ice) in the field immediately after collection, were transferred to a freezer (≤−10°C) for storage until shipment, and were then shipped frozen to EPL Bio Analytical Services (EPL BAS, Niantic, IL, USA) for composition analysis.

### Composition analysis

Forage samples were analyzed for proximates, fiber, and minerals [crude protein, crude fat, crude fiber, acid detergent fiber (ADF), neutral detergent fiber (NDF), ash, carbohydrates, calcium, and phosphorus]. Grain samples were analyzed for proximates and fiber [crude protein, crude fat, crude fiber, ADF, NDF, total dietary fiber (TDF), ash, and carbohydrates], minerals (calcium, copper, iron, magnesium, manganese, phosphorus, potassium, sodium, and zinc), fatty acids [lauric acid (C12:0), myristic acid (C14:0), palmitic acid (C16:0), palmitoleic acid (C16:1), heptadecanoic acid (C17:0), heptadecenoic acid (C17:1), stearic acid (C18:0), oleic acid (C18:1), linoleic acid (C18:2), α-linolenic acid (C18:3), arachidic acid (C20:0), eicosenoic acid (C20:1), eicosadienoic acid (C20:2), behenic acid (C22:0), and lignoceric acid (C24:0)], amino acids (alanine, arginine, aspartic acid, cystine, glutamic acid, glycine, histidine, isoleucine, leucine, lysine, methionine, phenylalanine, proline, serine, threonine, tryptophan, tyrosine, and valine), vitamins [β-carotene, vitamin B1 (thiamine), vitamin B2 (riboflavin), vitamin B3 (niacin), vitamin B5 (pantothenic acid), vitamin B6 (pyridoxine), vitamin B9 (folic acid), α-tocopherol, β-tocopherol, γ-tocopherol, and δ-tocopherol], as well as secondary metabolites and anti-nutrients (*p*-coumaric acid, ferulic acid, furfural, inositol, phytic acid, raffinose, and trypsin inhibitor). Forage samples were analyzed for the proximates, fiber, and minerals (Supplemental Information Table S2) by EPL BAS using Good Laboratory Practices (GLP) validated methods, as described previously.^18–20^ Grain samples were analyzed for the proximates, fiber, minerals, fatty acids amino acids, vitamins, and secondary metabolites and anti-nutrients (Supplemental Information Tables S3-S8) by EPL BAS using Good Laboratory Practices (GLP) validated methods, as described previously.^18–20^

### Statistical Analysis: Agronomic Assessment

Statistical analysis was conducted to compare the agronomic endpoints from DP915635 maize and control maize using SAS software, Version 9.4 (SAS Institute Inc., Cary, NC, USA). Endpoints were assessed for uniformity and were analyzed using across-site mixed model analysis (12 endpoints) or the generalized Cochran-Mantel-Haenszel (CMH) test (three endpoints), depending on if <50% or >50% of the sites had uniform data, respectively, as described previously.^17^ Three agronomic endpoints that did not meet criteria for minimum levels of non-uniformity were not statistically analyzed (Supplemental Information Table S1). The false discovery rate (FDR) method^21,22^ was used to control for false-positive outcomes since an adjusted P-value > 0.05 signifies that the observed statistically significant difference is likely a false positive, as described previously.^17^ Statistically significant agronomic endpoints were also assessed for biological relevance by comparing the individual values from DP915635 maize to the reference range (which is the range of all individual values across sites from all non-GM reference maize lines grown concurrently).

### Statistical Analysis: Composition Assessment

Statistical analysis was conducted to compare the composition from DP915635 maize and control maize using SAS software, Version 9.4. Composition analytes were analyzed using across-site mixed model analysis or using Fisher’s exact test, depending on the number of samples below the lower limit of quantification (LLOQ), as described previously.^17^ If 100% of samples from both GM and the control maize were below the LLOQ, then statistical analyses were not performed. The FDR method was used to control for false positives, and biological relevance of statistical differences was assessed by comparing DP915635 maize to one or more reference ranges (i.e., tolerance intervals, literature ranges, and in-study reference ranges, as described previously).^17^ Tolerance intervals were derived from proprietary accumulated data from 31 multi-site maize field studies conducted between 2003 and 2018. These studies consisted of a total of 167 non-GM commercial reference maize lines and 171 unique environments representative of commercial maize-growing regions in the United States, Canada, Chile, Brazil, and Argentina. Literature ranges were generated from relevant crop composition data obtained from published literature.^19,23–27^

## Results and Discussion

### Agronomic Assessment

The agronomic characteristics that were evaluated included early stand count, days to flowering, pollen viability, plant height, days to maturity, lodging, final stand count, dropped ears, yield, harvest grain moisture, and 100-kernel weight. Dropped ears and pollen shape and color at 120 min were not included in the statistical analysis because they did not meet the minimum levels of non-uniformity (Supplemental Information Table S1). The mean number of dropped ears for both DP915635 and control maize was 0.0. Pollen shape at 120 min was 98–99% of pollen with collapsed walls for both DP915635 and control maize. Greater than 99% of the pollen was yellow in color at 120 min for both DP915635 and control maize. Similar results have been observed previously for these agronomic characteristics and time points.^17,28^

No statistical differences were identified between DP915635 maize and control maize in the across-site analysis for pollen viability (pollen shape and color at 0, 30, and 60 min), plant height, days to maturity, lodging, final stand count, harvest grain moisture, yield, and 100-kernel weight (Supplemental Information Table S1). A statistically significant difference in the early stand count (P-value = .00541; FDR adjusted P-value = .0812) and days to flowering (P-value = .0279; FDR adjusted P-value = .209) was observed for DP915635 maize compared with control maize (Table 2). The FDR adjusted P-values for early stand count and days to flowering were not significant, indicating these were likely false positives. For early stand count, all of the DP915635 maize values were within the reference range (Table 2), indicating that the observed difference is not biologically relevant (i.e, within the range of natural variation for commercial non-GM maize). For days to flowering, 37 of 40 DP915635 values (with 3 values above the upper reference range) were within the reference data range. The minor differences observed for early stand count and days to flowering are unlikely to result in biologically relevant changes in DP915635 maize plants that could alter characteristics relevant for assessing environmental risk (weediness potential or survivability). The results obtained from this field study demonstrate that the agronomic endpoints of DP915635 maize are comparable to those derived from non-GM maize.

**Table 2.** Across-site analysis results for agronomic characteristics that had statistically significant differences between DP915635 maize and non-GM near-isoline control maize (control).

Agronomic Characteristic	Reported Statistics^a^	Control Maize	DP915635 Maize	Reference Range^c^
Early Stand (count/m^2^)	Mean	5.8	5.9	4.1–6.5
	Range	4.6–6.4	4.7–6.4	
	Confidence Interval	5.5–6.2	5.6–6.3	
	Adjusted P-Value	–	0.0812	
	P-Value	–	<0.00541*	
Days to Flowering (days)	Mean	63.9	63.5	54–74
	Range	55–75	56–76	
	Confidence Interval	59.5–68.3	59.1–67.9	
	Adjusted P-Value^b^	–	0.209	
	P-Value^b^	–	0.0279*	

^a^The across-site statistical analysis for each agronomic characteristic included results from all ten field sites with four replicate blocks at each site.

^b^P-values < 0.05 are denoted by *. Adjusted P-values were used to control for false positive outcomes across all agronomic characteristics analyzed using linear mixed models.

^c^Reference ranges were obtained from four non-GM commercial maize lines grown at each site in the study.

### Composition Assessment

Nutrient composition data were generated for a total of 79 analytes, including 9 analytes that were measured in forage (proximates, fiber, and minerals) and 70 analytes that were measured in grain (proximates and fiber, minerals, fatty acids, amino acids, vitamins, and secondary metabolites and anti-nutrients). No statistically significant differences were observed between DP915635 maize and control maize for the nine forage analytes (Supplemental Information Table S2). For grain, across-site comparisons were conducted for a total of 64 analytes (Supplemental Information Tables S3–S8). Two of these analytes (14:0 myristic acid and δ-tocopherol) did not meet the criteria for sufficient quantities of observations above the LLOQ and were therefore subjected to Fisher’s exact test (Supplemental Information Table S9). Six analytes [12:0 lauric acid, 17:1 heptadecenoic acid, 20:2 eicosadienoic acid, vitamin B2 (Riboflavin), β-tocopherol, and furfural] were not statistically analyzed because values were all below the LLOQ (Supplemental Information Table S9). A statistically significant difference was observed in the across-site analysis between DP915635 maize and control maize for seven grain analytes: 16:1 palmitoleic acid, 18:0 stearic acid, 18:1 oleic acid, 18:2 linoleic acid, 24:0 lignoceric acid, methionine, and α-tocopherol (Table 3). The FDR adjusted P-values were not significant for these analytes, indicating that the observed differences were likely false positives. Furthermore, all individual values for these analytes were within the tolerance interval, literature range, and/or in-study reference range, indicating that DP915635 maize is within the range of natural variation for these analytes and that the statistical differences are not biologically meaningful.

**Table 3.** Across-site analysis results for composition analytes that had statistically significant differences between DP915635 maize and non-GM near-isoline control maize (control).

Composition Analyte^a^	DP915635 Mean (Range)	Control Mean (Range)	P-value (Adjusted P-value)^b^	Reference Maize Range^c^	Tolerance Interval^d^	Literature Range^e^
Palmitoleic acid (C16:1)	0.122 (0.0999–0.140)	0.119 (0.0940–0.145)	0.0178* (0.289)	0.0911–0.184	0–0.435	NQ − 0.67
Stearic acid (C18:0)	2.02 (1.75–2.64)	1.98 (1.74–2.44)	0.0297* (0.301)	1.73–2.92	1.32–3.69	NQ − 4.9
Oleic acid (C18:1)	2.38 (22.0–25.0)	24.1 (22.2–25.6)	0.00864* (0.204)	21.0–32.2	16.9–38.4	16.38–42.81
Linoleic acid (C18:2)	5.92 (56.9–61.4)	58.8 (56.6–61.4)	0.00822* (0.204)	49.2–61.5	31.9–65.3	13.1–67.68
Lignoceric acid (C24:0)	0.294 (0.261–0.367)	0.301 (0.270–0.376)	0.0203* (0.289)	0.250–0.451	0–0.622	NQ − 0.91
Methionine	0.200 (0.147–0.260)	0.214 (0.165–0.292)	0.0287* (0.301)	0.151–0.255	0.106–0.315	0.10–0.47
α-tocopherol	3.97 (<0.500^f^ − 9.97)	3.67 (<0.500^f^ − 9.83)	0.00147* (0.104)	<0.500^f^ − 25.2	0–22.9	NQ − 68.67

^a^The across-site statistical analysis for each analyte included results from all eight field sites with four replicate blocks at each site.

^b^P-values < 0.05 are denoted by *. Adjusted P-values were used to control for false positive outcomes across all analytes analyzed using linear mixed models.

^c^Reference ranges were obtained from four non-GM commercial maize lines grown at each site in the study.

^d^Tolerance intervals were derived from Corteva Agriscience’s™ proprietary accumulated data from commercial non-GM maize lines.

^e^Literature ranges were obtained from published literature.^19,23–27^ NQ; not detectable: one or more assay values in the published literature references were below the lower limit of quantification (LLOQ) and were not quantified.

^f^< LLOQ, one or more sample values were below the assay LLOQ.

## Discussion

DP915635 was modified to contain an insect protection trait (IPD079Ea protein), a herbicide tolerance trait (PAT protein), and a selectable marker (PMI protein). Maize biology is well documented, and there is consensus that maize is a highly domesticated crop that is unlikely to survive outside of cultivation.^29^ Furthermore, maize is not considered a weedy or invasive species.^29,30^ As part of the regulatory requirements for GM crop cultivation, a large-scale, multi-site agronomic assessment was conducted to compare the agronomic characteristics of DP915635 to non-GM maize. The agronomic assessment informs the environmental risk assessment for cultivation of a GM crop (e.g., assessment of potential for weediness, gene flow, survival, etc.), and DP915635 maize has been shown to be comparable to non-GM maize. It is therefore concluded that DP915635 maize is unlikely to pose an increased risk to the environment compared with non-GM maize, based on knowledge about the intended traits, the basic biology of maize, and results of the agronomic assessment.

As demonstrated by results from a large multi-site field study, the composition of DP915635 maize is comparable to that of non-GM maize, with few statistically significant differences in analytes detected. In the limited number of composition analytes where statistically significant differences were detected, the differences were not significant following FDR-adjustment, indicating that they are likely false positives. Furthermore, none of the statistically significant differences were determined to be biologically relevant because the values of DP915635 maize for those analytes were within established ranges for non-GM maize (tolerance intervals, literature range, and study reference range). Crop composition studies have been required for the safety assessment of GM crops since 1993^31^; however, scores of composition assessments with GM events in numerous crop species have concluded equivalence between the GM crops and their non-GM counterparts.^32^ This substantial body of evidence that has been generated over the past 30 years calls into question the scientific merit of a routine requirement for composition assessments with GM crops and indicates that these assessments should only be required for GM events where there is a plausible scientific hypothesis for how the genetic modification could result in a change in composition that would impact food or feed safety. Knowledge of the intended trait and the experience accumulated from nearly 30 years of GM crop cultivation and safety assessment should be leveraged to inform the safety assessment. The intended traits included in DP915635 maize confer insect protection and herbicide tolerance, which pose no plausible scientific hypothesis for a change in the composition of DP915635 maize compared with that of non-GM maize. The mode of action of the IPD079Ea protein (binding to receptors in the midgut epithelial cells of WCR) is understood, and the PAT and PMI proteins are well known and widely used, as they have been approved for commercial use in multiple events and crops.^33–36^ These results add to the weight of evidence supporting the conclusion that DP915635 maize is as safe and nutritious as non-GM maize for food, feed, and the environment.

## Supplementary Material

Supplemental Material
