# Supplementary material for: Genetically modified DP915635 maize is agronomically and compositionally comparable to non-genetically modified maize
Source: GM Crops Food. 2023 May 4;14(1):1–8. doi: 10.1080/21645698.2023.2208997 (PMC10161957; doi:10.1080/21645698.2023.2208997)
Supplement: Supplemental Material [file KGMC_A_2208997_SM7150.docx]

**Genetically modified DP915635 maize is agronomically and compositionally comparable to non-genetically modified maize**

Jennifer A. Anderson*, James Mickelson, Brandon J. Fast, Nathan Smith, Robert C. Pauli, and Carl Walker

Corteva Agriscience™, 7300 NW 62nd Avenue, Johnston, IA 50131, USA

*Corresponding Author

Corteva Agriscience™

Johnston, IA 50131, USA

[Jennifer.Anderson@corteva.com](mailto:Jennifer.Anderson@corteva.com)

(515) 535-3730 (phone)

**Supplemental Information Table 1:** Mean and range (minimum and maximum individual values) of all agronomic characteristics from DP915635 maize and non-GM near-isoline control maize (control). In-study reference ranges were obtained from the four non-GM commercial maize lines grown at each site.

| **Agronomic Characteristic** | **Reported Statistics** | **Control Maize** | **DP915635 Maize** | **Reference Data Range** |
| --- | --- | --- | --- | --- |
| Early Stand (count/m^2^) | Mean | 5.8 | 5.9 | 4.1 - 6.5 |
|  | Range | 4.6 - 6.4 | 4.7 - 6.4 |  |
|  | Confidence Interval | 5.5 - 6.2 | 5.6 - 6.3 |  |
|  | Adjusted P-Value | -- | 0.0812 |  |
|  | P-Value | -- | 0.00541^*^ |  |
| Days to Flowering (days) | Mean | 63.9 | 63.5 | 54 - 74 |
|  | Range | 55 - 75 | 56 - 76 |  |
|  | Confidence Interval | 59.5 - 68.3 | 59.1 - 67.9 |  |
|  | Adjusted P-Value | -- | 0.209 |  |
|  | P-Value | -- | 0.0279^*^ |  |
| Pollen Viability-Shape, 0 minutes  (% of pollen with collapsed walls) | Mean | 5.0 | 5.8 | 0 - 30 |
|  | Range | 0 - 20 | 0 - 20 |  |
|  | Confidence Interval | 1.0 - 9.0 | 1.8 - 9.8 |  |
|  | Adjusted P-Value | -- | 0.466 |  |
|  | P-Value | -- | 0.310 |  |
| Pollen Viability-Shape, 30 minutes  (% of pollen with collapsed walls) | Mean | 62.3 | 64.8 | 0 - 100 |
|  | Range | 0 - 100 | 0 - 100 |  |
|  | Confidence Interval | 38.2 - 86.3 | 40.7 - 88.8 |  |
|  | Adjusted P-Value | -- | 0.388 |  |
|  | P-Value | -- | 0.181 |  |
| Pollen Viability-Shape, 60 minutes  (% of pollen with collapsed walls) | Mean | 84.4 | 88.8 | 0 - 100 |
|  | Range | 5 - 100 | 15 - 100 |  |
|  | Confidence Interval | NA | NA |  |
|  | Adjusted P-Value | -- | 0.388 |  |
|  | P-Value | -- | 0.156 |  |
| Pollen Viability-Shape, 120 minutes  (% of pollen with collapsed walls) | Mean | 98.6 | 99.5 | 90 - 100 |
|  | Range | 80 - 100 | 90 - 100 |  |
|  | Confidence Interval | NA | NA |  |
|  | Adjusted P-Value | -- | NA |  |
|  | P-Value | -- | NA |  |
| Pollen Viability-Color, 0 minutes  (% of pollen yellow in color) | Mean | 5.4 | 6.8 | 0 - 50 |
|  | Range | 0 - 20 | 0 - 20 |  |
|  | Confidence Interval | 1.5 - 9.3 | 2.9 - 10.6 |  |
|  | Adjusted P-Value | -- | 0.282 |  |
|  | P-Value | -- | 0.0752 |  |
| Pollen Viability-Color, 30 minutes  (% of pollen yellow in color) | Mean | 67.0 | 71.4 | 0 - 100 |
|  | Range | 0 - 100 | 0 - 100 |  |
|  | Confidence Interval | 34.4 - 92.3 | 39.0 - 94.7 |  |
|  | Adjusted P-Value | -- | 0.282 |  |
|  | P-Value | -- | 0.0649 |  |
| Pollen Viability-Color, 60 minutes  (% of pollen yellow in color) | Mean | 83.5 | 87.3 | 0 - 100 |
|  | Range | 0 - 100 | 10 - 100 |  |
|  | Confidence Interval | NA | NA |  |
|  | Adjusted P-Value | -- | 0.391 |  |
|  | P-Value | -- | 0.220 |  |
| Pollen Viability-Color, 120 minutes  (% of pollen yellow in color) | Mean | 99.4 | 99.9 | 95 - 100 |
|  | Range | 90 - 100 | 95 - 100 |  |
|  | Confidence Interval | NA | NA |  |
|  | Adjusted P-Value | -- | NA |  |
|  | P-Value | -- | NA |  |

Supplemental Information Table 1 (continued)

| **Agronomic Characteristic** | **Reported Statistics** | **Control Maize** | | | **DP915635 Maize** | **Reference Data Range** |
| --- | --- | --- | --- | --- | --- | --- |
| Plant Height (cm) | Mean | 247.3 | | | 247.3 | 161.8 - 291.6 |
|  | Range | 181.8 - 301.0 | | | 183.2 - 300.4 |  |
|  | Confidence Interval | 223.4 - 271.2 | | | 223.4 - 271.2 |  |
|  | Adjusted P-Value | -- | | | 0.999 |  |
|  | P-Value | -- | | | 0.999 |  |
| Days to Maturity (days) | Mean | 126.3 | | | 126.4 | 91 - 158 |
|  | Range | 95 - 158 | | | 93 - 155 |  |
|  | Confidence Interval | 114.3 - 138.3 | | | 114.4 - 138.4 |  |
|  | Adjusted P-Value | -- | | | 0.969 |  |
|  | P-Value | -- | | | 0.839 |  |
| Lodging (%) | Mean | 1.5 | | | 2.1 | 0.0 - 9.9 |
|  | Range | 0.0 - 8.6 | | | 0.0 - 12.3 |  |
|  | Confidence Interval | NA | | | NA |  |
|  | Adjusted P-Value | -- | | | 0.391 |  |
|  | P-Value | -- | | | 0.235 |  |
| Final Stand Count (count/m^2^) | Mean | 5.8 | | | 5.8 | 4.2 - 6.4 |
|  | Range | 4.6 - 6.4 | | | 4.7 - 6.4 |  |
|  | Confidence Interval | 5.5 - 6.1 | | | 5.5 - 6.2 |  |
|  | Adjusted P-Value | -- | | | 0.344 |  |
|  | P-Value | -- | | | 0.115 |  |
| Dropped Ears (count) | Mean | 0.0 | | | 0.0 | 0 - 2 |
|  | Range | 0 - 0 | | | 0 - 1 |  |
|  | Confidence Interval | NA | | | NA |  |
|  | Adjusted P-Value | -- | | | NA |  |
|  | P-Value | -- | | | NA |  |
| Harvest Grain Moisture (%) | Mean | 21.0 | | | 21.1 | 11.0 - 30.5 |
|  | Range | 11.3 - 37.0 | | | 11.2 - 32.8 |  |
|  | Confidence Interval | 17.1 - 24.9 | | | 17.2 - 25.0 |  |
|  | Adjusted P-Value | -- | | | 0.855 |  |
|  | P-Value | -- | | | 0.684 |  |
| Yield (bu/A) | Mean | 163.6 | | | 166.8 | 38 - 273 |
|  | Range | 54 - 261 | | | 40 - 255 |  |
|  | Confidence Interval | 122.7 - 204.5 | | | 125.9 - 207.7 |  |
|  | Adjusted P-Value | -- | | | 0.580 |  |
|  | P-Value | -- | | | 0.425 |  |
| 100-Kernel Weight (g) | Mean | 32.9 | | | 32.9 | 21.3 - 46.5 |
|  | Range | 23.7 - 40.9 | | | 20.8 - 42.6 |  |
|  | Confidence Interval | 29.7 - 36.1 | | | 29.7 - 36.1 |  |
|  | Adjusted P-Value | -- | | | 0.999 |  |
|  | P-Value | -- | | | 0.960 |  |
| Note: Not applicable (NA); mixed model analysis was not performed. | | | | |  |  |
| ^*^ A statistically significant difference (P-value < 0.05) was observed. | | | |  |  |  |

Supplemental Information Table 2: Mean and range (minimum and maximum individual values) of proximates, fiber, and minerals in forage from DP915635 maize and non-GM near-isoline control maize (control). Tolerance intervals were derived from Corteva Agriscience’s™ proprietary accumulated data from commercial non-GM maize lines. Literature ranges were obtained from published literature. In-study reference ranges were obtained from the four non-GM commercial maize lines grown at each site.

| **Analyte** | **Reported Statistics** | **Control Maize** | **DP915635 Maize** | **Tolerance Interval** | **Literature Range** | **Reference Data Range** |
| --- | --- | --- | --- | --- | --- | --- |
| Crude Protein | Mean | 7.22 | 7.22 | 3.64 - 12.3 | 2.37 - 16.32 | 3.91 - 10.4 |
|  | Range | 3.55 - 9.43 | 5.08 - 9.29 |  |  |  |
|  | Confidence Interval | 6.24 - 8.19 | 6.24 - 8.20 |  |  |  |
|  | Adjusted P-Value | -- | 0.996 |  |  |  |
|  | P-Value | -- | 0.983 |  |  |  |
| Crude Fat | Mean | 3.65 | 3.73 | 0.822 - 6.42 | NQ - 6.755 | 2.11 - 5.43 |
|  | Range | 2.00 - 6.08 | 2.33 - 4.82 |  |  |  |
|  | Confidence Interval | 3.25 - 4.06 | 3.32 - 4.14 |  |  |  |
|  | Adjusted P-Value | -- | 0.996 |  |  |  |
|  | P-Value | -- | 0.636 |  |  |  |
| Crude Fiber | Mean | 23.1 | 22.6 | 13.8 - 31.0 | 12.5 - 42 | 12.6 - 28.8 |
|  | Range | 18.3 - 30.4 | 15.1 - 30.0 |  |  |  |
|  | Confidence Interval | 21.1 - 25.2 | 20.5 - 24.6 |  |  |  |
|  | Adjusted P-Value | -- | 0.996 |  |  |  |
|  | P-Value | -- | 0.416 |  |  |  |
| ADF | Mean | 28.0 | 27.2 | 15.7 - 39.9 | 5.13 - 47.39 | 11.4 - 35.8 |
|  | Range | 20.1 - 35.8 | 15.7 - 36.0 |  |  |  |
|  | Confidence Interval | 25.3 - 30.7 | 24.6 - 29.9 |  |  |  |
|  | Adjusted P-Value | -- | 0.996 |  |  |  |
|  | P-Value | -- | 0.367 |  |  |  |
| NDF | Mean | 45.6 | 46.1 | 28.6 - 63.2 | 18.30 - 67.80 | 26.8 - 57.1 |
|  | Range | 33.2 - 55.2 | 31.2 - 58.3 |  |  |  |
|  | Confidence Interval | 41.9 - 49.2 | 42.5 - 49.8 |  |  |  |
|  | Adjusted P-Value | -- | 0.996 |  |  |  |
|  | P-Value | -- | 0.586 |  |  |  |
| Ash | Mean | 4.21 | 4.16 | 2.43 - 9.36 | 0.66 - 13.20 | 2.55 - 7.24 |
|  | Range | 3.03 - 5.88 | 2.84 - 5.80 |  |  |  |
|  | Confidence Interval | 3.68 - 4.74 | 3.63 - 4.69 |  |  |  |
|  | Adjusted P-Value | -- | 0.996 |  |  |  |
|  | P-Value | -- | 0.700 |  |  |  |
| Carbohydrates | Mean | 84.9 | 84.9 | 76.8 - 91.3 | 73.3 - 92.9 | 78.7 - 89.0 |
|  | Range | 80.8 - 91.2 | 81.4 - 87.6 |  |  |  |
|  | Confidence Interval | 83.4 - 86.4 | 83.4 - 86.4 |  |  |  |
|  | Adjusted P-Value | -- | 0.996 |  |  |  |
|  | P-Value | -- | 0.982 |  |  |  |
| Calcium | Mean | 0.203 | 0.206 | 0.0755 - 0.530 | 0.04 - 0.58 | 0.0848 - 0.358 |
|  | Range | 0.0677 - 0.354 | 0.0902 - 0.315 |  |  |  |
|  | Confidence Interval | 0.165 - 0.242 | 0.168 - 0.244 |  |  |  |
|  | Adjusted P-Value | -- | 0.996 |  |  |  |
|  | P-Value | -- | 0.856 |  |  |  |
| Phosphorus | Mean | 0.244 | 0.239 | 0.0899 - 0.433 | 0.07 - 0.55 | 0.111 - 0.385 |
|  | Range | 0.149 - 0.376 | 0.133 - 0.332 |  |  |  |
|  | Confidence Interval | 0.210 - 0.278 | 0.205 - 0.273 |  |  |  |
|  | Adjusted P-Value | -- | 0.996 |  |  |  |
|  | P-Value | -- | 0.613 |  |  |  |

Note: Proximate, fiber, and mineral unit of measure is % dry weight. Not quantified (NQ); one or more assay values in the published literature references were below the lower limit of quantification (LLOQ) and were not quantified. Literature range (AFSI, 2019; Codex Alimentarius Commission, 2019; Cong et al., 2015; Lundry et al., 2013; OECD, 2002; Watson, 1982).

Supplemental Information Table 3: Mean and range (minimum and maximum individual values) of proximates and fiber in grain from DP915635 maize and non-GM near-isoline control maize (control). Tolerance intervals were derived from Corteva Agriscience’s™ proprietary accumulated data from commercial non-GM maize lines. Literature ranges were obtained from published literature. In-study reference ranges were obtained from the four non-GM commercial maize lines grown at each site.

| **Analyte** | **Reported Statistics** | **Control Maize** | **DP915635 Maize** | **Tolerance Interval** | **Literature Range** | **Reference Data Range** |
| --- | --- | --- | --- | --- | --- | --- |
| Moisture | Mean | 21.5 | 21.4 | 3.92 - 37.8 | 5.1 - 40.7 | 9.89 - 32.4 |
|  | Range | 10.6 - 31.9 | 10.0 - 32.1 |  |  |  |
|  | Confidence Interval | 17.0 - 26.0 | 16.9 - 25.9 |  |  |  |
|  | Adjusted P-Value | -- | 0.996 |  |  |  |
|  | P-Value | -- | 0.643 |  |  |  |
| Crude Protein | Mean | 9.71 | 9.64 | 6.63 - 13.2 | 5.72 - 17.26 | 7.05 - 11.0 |
|  | Range | 7.77 - 11.5 | 8.09 - 11.9 |  |  |  |
|  | Confidence Interval | 8.99 - 10.4 | 8.91 - 10.4 |  |  |  |
|  | Adjusted P-Value | -- | 0.996 |  |  |  |
|  | P-Value | -- | 0.598 |  |  |  |
| Crude Fat | Mean | 3.60 | 3.68 | 2.38 - 5.91 | 1.363 - 7.830 | 1.95 - 5.31 |
|  | Range | 2.92 - 5.07 | 2.81 - 4.72 |  |  |  |
|  | Confidence Interval | 3.40 - 3.79 | 3.49 - 3.88 |  |  |  |
|  | Adjusted P-Value | -- | 0.996 |  |  |  |
|  | P-Value | -- | 0.495 |  |  |  |
| Crude Fiber | Mean | 2.42 | 2.41 | 1.58 - 3.54 | 0.49 - 5.5 | 1.94 - 3.08 |
|  | Range | 2.08 - 2.78 | 1.94 - 2.77 |  |  |  |
|  | Confidence Interval | 2.31 - 2.53 | 2.30 - 2.52 |  |  |  |
|  | Adjusted P-Value | -- | 0.996 |  |  |  |
|  | P-Value | -- | 0.763 |  |  |  |
| ADF | Mean | 4.19 | 4.24 | 2.67 - 6.15 | 1.41 - 11.34 | 2.53 - 5.69 |
|  | Range | 3.47 - 4.90 | 3.26 - 5.41 |  |  |  |
|  | Confidence Interval | 3.91 - 4.47 | 3.96 - 4.52 |  |  |  |
|  | Adjusted P-Value | -- | 0.996 |  |  |  |
|  | P-Value | -- | 0.639 |  |  |  |
| NDF | Mean | 10.8 | 10.9 | 7.57 - 18.1 | 4.28 - 24.30 | 7.98 - 17.9 |
|  | Range | 8.83 - 15.7 | 9.24 - 19.0 |  |  |  |
|  | Confidence Interval | 9.60 - 12.1 | 9.71 - 12.2 |  |  |  |
|  | Adjusted P-Value | -- | 0.996 |  |  |  |
|  | P-Value | -- | 0.757 |  |  |  |
| Total Dietary Fiber | Mean | 8.73 | 8.77 | 3.14 - 20.6 | 5.78 - 35.31 | 6.99 - 12.1 |
|  | Range | 7.63 - 10.3 | 7.73 - 10.0 |  |  |  |
|  | Confidence Interval | 8.41 - 9.05 | 8.45 - 9.09 |  |  |  |
|  | Adjusted P-Value | -- | 0.996 |  |  |  |
|  | P-Value | -- | 0.746 |  |  |  |
| Ash | Mean | 1.34 | 1.33 | 0.959 - 1.78 | 0.616 - 6.282 | 0.916 - 1.48 |
|  | Range | 0.861 - 1.63 | 0.862 - 1.52 |  |  |  |
|  | Confidence Interval | 1.23 - 1.46 | 1.21 - 1.44 |  |  |  |
|  | Adjusted P-Value | -- | 0.996 |  |  |  |
|  | P-Value | -- | 0.290 |  |  |  |
| Carbohydrates | Mean | 85.2 | 85.4 | 80.6 - 88.5 | 77.4 - 89.7 | 82.4 - 88.8 |
|  | Range | 82.9 - 87.8 | 82.3 - 87.5 |  |  |  |
|  | Confidence Interval | 84.3 - 86.1 | 84.5 - 86.3 |  |  |  |
|  | Adjusted P-Value | -- | 0.497 |  |  |  |
|  | P-Value | -- | 0.0754 |  |  |  |

Note: Proximate and fiber unit of measure is % dry weight, with the exception of moisture (%). Literature range (AFSI, 2019; Codex Alimentarius Commission, 2019; Cong et al., 2015; Lundry et al., 2013; OECD, 2002; Watson, 1982).

Supplemental Information Table 4: Mean and range (minimum and maximum individual values) of fatty acids in grain from DP915635 maize and non-GM near-isoline control maize (control). Tolerance intervals were derived from Corteva Agriscience’s™ proprietary accumulated data from commercial non-GM maize lines. Literature ranges were obtained from published literature. In-study reference ranges were obtained from the four non-GM commercial maize lines grown at each site.

| **Analyte** | | **Reported Statistics** | **Control Maize** | **DP915635 Maize** | **Tolerance Interval** | | **Literature Range** | **Reference Data Range** | |  |
| --- | --- | --- | --- | --- | --- | --- | --- | --- | --- | --- |
| Lauric Acid (C12:0) | Mean | | <LLOQ^a^ | <LLOQ^a^ | 0 - 0.423^b^ | NQ - 0.698 | | | <LLOQ^a^ | |
|  | Range | | <LLOQ^a^ | <LLOQ^a^ |  |  |  |  |  |  |
|  | Confidence Interval | | NA | NA |  |  |  |  |  |  |
|  | Adjusted P-Value | | -- | NA |  |  |  |  |  |  |
|  | P-Value | | -- | NA |  |  |  |  |  |  |
| Myristic Acid (C14:0) | Mean | | <LLOQ^a^ | 0.0420 | 0 - 0.267^b^ | NQ - 0.288 | | | 0.0319 - 0.0994 | |
|  | Range | | <LLOQ^a^ | 0.0353 - 0.101 |  |  |  |  |  |  |
|  | Confidence Interval | | NA | NA |  |  |  |  |  |  |
|  | Adjusted P-Value | | -- | NA |  |  |  |  |  |  |
|  | P-Value | | -- | NA |  |  |  |  |  |  |
| Palmitic Acid (C16:0) | Mean | | 11.8 | 11.7 | 9.45 - 24.5 | 6.81 - 39.0 | | | 10.8 - 14.7 | |
|  | Range | | 11.3 - 13.0 | 11.2 - 12.7 |  |  |  |  |  |  |
|  | Confidence Interval | | 11.4 - 12.1 | 11.4 - 12.1 |  |  |  |  |  |  |
|  | Adjusted P-Value | | -- | 0.996 |  |  |  |  |  |  |
|  | P-Value | | -- | 0.451 |  |  |  |  |  |  |
| Palmitoleic Acid (C16:1) | Mean | | 0.119 | 0.122 | 0 - 0.435 | NQ - 0.67 | | | 0.0911 - 0.184 | |
|  | Range | | 0.0940 - 0.145 | 0.0999 - 0.140 |  |  |  |  |  |  |
|  | Confidence Interval | | 0.111 - 0.127 | 0.114 - 0.131 |  |  |  |  |  |  |
|  | Adjusted P-Value | | -- | 0.289 |  |  |  |  |  |  |
|  | P-Value | | -- | **0.0178^*^** |  |  |  |  |  |  |
| Heptadecanoic Acid (C17:0) | Mean | | 0.0954 | 0.0937 | 0 - 0.225 | NQ - 0.203 | | | 0.0361 - 0.156 | |
|  | Range | | 0.0393 - 0.116 | 0.0387 - 0.112 |  |  |  |  |  |  |
|  | Confidence Interval | | 0.0853 - 0.106 | 0.0835 - 0.104 |  |  |  |  |  |  |
|  | Adjusted P-Value | | -- | 0.608 |  |  |  |  |  |  |
|  | P-Value | | -- | 0.103 |  |  |  |  |  |  |
| Heptadecenoic Acid (C17:1) | Mean | | <LLOQ^a^ | <LLOQ^a^ | 0 - 0.135^b^ | NQ - 0.131 | | | 0.0323 - 0.0990 | |
|  | Range | | <LLOQ^a^ | <LLOQ^a^ |  |  |  |  |  |  |
|  | Confidence Interval | | NA | NA |  |  |  |  |  |  |
|  | Adjusted P-Value | | -- | NA |  |  |  |  |  |  |
|  | P-Value | | -- | NA |  |  |  |  |  |  |
| Stearic Acid (C18:0) | Mean | | 1.98 | 2.02 | 1.32 - 3.69 | NQ - 4.9 | | | 1.73 - 2.92 | |
|  | Range | | 1.74 - 2.44 | 1.75 - 2.64 |  |  |  |  |  |  |
|  | Confidence Interval | | 1.80 - 2.16 | 1.84 - 2.20 |  |  |  |  |  |  |
|  | Adjusted P-Value | | -- | 0.301 |  |  |  |  |  |  |
|  | P-Value | | -- | **0.0297^*^** |  |  |  |  |  |  |
| Oleic Acid (C18:1) | Mean | | 24.1 | 23.8 | 16.9 - 38.4 | 16.38 - 42.81 | | | 21.0 - 32.2 | |
|  | Range | | 22.2 - 25.6 | 22.0 - 25.0 |  |  |  |  |  |  |
|  | Confidence Interval | | 23.6 - 24.5 | 23.3 - 24.2 |  |  |  |  |  |  |
|  | Adjusted P-Value | | -- | 0.204 |  |  |  |  |  |  |
|  | P-Value | | -- | **0.00864^*^** |  |  |  |  |  |  |
| Linoleic Acid (C18:2) | Mean | | 58.8 | 59.2 | 31.9 - 65.3 | 13.1 - 67.68 | | | 49.2 - 61.5 | |
|  | Range | | 56.6 - 61.4 | 56.9 - 61.4 |  |  |  |  |  |  |
|  | Confidence Interval | | 58.0 - 59.7 | 58.3 - 60.0 |  |  |  |  |  |  |
|  | Adjusted P-Value | | -- | 0.204 |  |  |  |  |  |  |
|  | P-Value | | -- | **0.00822^*^** |  |  |  |  |  |  |
| α-Linolenic Acid (C18:3) | Mean | | 1.74 | 1.73 | 0 - 2.06 | NQ - 2.33 | | | 1.46 - 2.31 | |
|  | Range | | 1.55 - 1.89 | 1.51 - 1.92 |  |  |  |  |  |  |
|  | Confidence Interval | | 1.68 - 1.81 | 1.66 - 1.79 |  |  |  |  |  |  |
|  | Adjusted P-Value | | -- | 0.996 |  |  |  |  |  |  |
|  | P-Value | | -- | 0.285 |  |  |  |  |  |  |

**Supplemental Information Table 4** **(continued)**

| **Analyte** | **Reported Statistics** | **Control Maize** | **DP915635 Maize** | **Tolerance Interval** | **Literature Range** | **Reference Data Range** |
| --- | --- | --- | --- | --- | --- | --- |
| Arachidic Acid (C20:0) | Mean | 0.372 | 0.371 | 0.296 - 0.850 | 0.267 - 1.2 | 0.328 - 0.510 |
|  | Range | 0.329 - 0.458 | 0.327 - 0.469 |  |  |  |
|  | Confidence Interval | 0.341 - 0.403 | 0.340 - 0.402 |  |  |  |
|  | Adjusted P-Value | -- | 0.996 |  |  |  |
|  | P-Value | -- | 0.511 |  |  |  |
| Eicosenoic Acid (C20:1) | Mean | 0.319 | 0.313 | 0 - 0.581 | NQ - 1.952 | 0.247 - 0.433 |
|  | Range | 0.278 - 0.423 | 0.265 - 0.332 |  |  |  |
|  | Confidence Interval | 0.308 - 0.331 | 0.302 - 0.325 |  |  |  |
|  | Adjusted P-Value | -- | 0.497 |  |  |  |
|  | P-Value | -- | 0.0620 |  |  |  |
| Eicosadienoic Acid (C20:2) | Mean | <LLOQ^a^ | <LLOQ^a^ | 0 - 0.825^b^ | NQ - 2.551 | <LLOQ^a^ |
|  | Range | <LLOQ^a^ | <LLOQ^a^ |  |  |  |
|  | Confidence Interval | NA | NA |  |  |  |
|  | Adjusted P-Value | -- | NA |  |  |  |
|  | P-Value | -- | NA |  |  |  |
| Behenic Acid (C22:0) | Mean | 0.232 | 0.232 | 0 - 0.430 | NQ - 0.5 | 0.168 - 0.330 |
|  | Range | 0.171 - 0.299 | 0.173 - 0.295 |  |  |  |
|  | Confidence Interval | 0.210 - 0.254 | 0.210 - 0.254 |  |  |  |
|  | Adjusted P-Value | -- | 0.996 |  |  |  |
|  | P-Value | -- | 0.994 |  |  |  |
| Lignoceric Acid (C24:0) | Mean | 0.301 | 0.294 | 0 - 0.622 | NQ - 0.91 | 0.250 - 0.451 |
|  | Range | 0.270 - 0.376 | 0.261 - 0.367 |  |  |  |
|  | Confidence Interval | 0.276 - 0.325 | 0.270 - 0.318 |  |  |  |
|  | Adjusted P-Value | -- | 0.289 |  |  |  |
|  | P-Value | -- | **0.0203^*^** |  |  |  |

Note: Fatty acid unit of measure is % total fatty acids. Not applicable (NA); mixed model analysis was not performed or confidence interval was not determined. Not quantified (NQ); one or more assay values in the published literature references were below the LLOQ and were not quantified. Literature range (AFSI, 2019; Codex Alimentarius Commission, 2019; Cong et al., 2015; Lundry et al., 2013; OECD, 2002; Watson, 1982).

^a^ < LLOQ, all fatty acid sample values in the current study were below the assay LLOQ. Statistical analysis was not performed for those analytes.

^b^ A historical reference data range was provided as tolerance interval was not calculated since the data did not meet the assumptions of any tolerance interval calculation method.

^*^ A statistically significant difference (P-Value < 0.05) was observed.

###### Supplemental Information Table 5: Mean and range (minimum and maximum individual values) of amino acids in grain from DP915635 maize and non-GM near-isoline control maize (control). Tolerance intervals were derived from Corteva Agriscience’s™ proprietary accumulated data from commercial non-GM maize lines. Literature ranges were obtained from published literature. In-study reference ranges were obtained from the four non-GM commercial maize lines grown at each site.

| **Analyte** | **Reported Statistics** | **Control Maize** | **DP915635 Maize** | **Tolerance Interval** | **Literature Range** | **Reference Data Range** |
| --- | --- | --- | --- | --- | --- | --- |
| Alanine | Mean | 0.720 | 0.719 | 0.453 - 1.07 | 0.40 - 1.48 | 0.465 - 0.865 |
|  | Range | 0.550 - 0.887 | 0.580 - 0.944 |  |  |  |
|  | Confidence Interval | 0.658 - 0.782 | 0.657 - 0.781 |  |  |  |
|  | Adjusted P-Value | -- | 0.996 |  |  |  |
|  | P-Value | -- | 0.956 |  |  |  |
| Arginine | Mean | 0.406 | 0.400 | 0.305 - 0.592 | 0.12 - 0.71 | 0.302 - 0.481 |
|  | Range | 0.339 - 0.469 | 0.326 - 0.467 |  |  |  |
|  | Confidence Interval | 0.384 - 0.428 | 0.378 - 0.422 |  |  |  |
|  | Adjusted P-Value | -- | 0.996 |  |  |  |
|  | P-Value | -- | 0.482 |  |  |  |
| Aspartic Acid | Mean | 0.610 | 0.614 | 0.415 - 0.895 | 0.30 - 1.21 | 0.412 - 0.758 |
|  | Range | 0.493 - 0.723 | 0.498 - 0.749 |  |  |  |
|  | Confidence Interval | 0.560 - 0.660 | 0.564 - 0.665 |  |  |  |
|  | Adjusted P-Value | -- | 0.996 |  |  |  |
|  | P-Value | -- | 0.714 |  |  |  |
| Cystine | Mean | 0.234 | 0.229 | 0.129 - 0.294 | 0.12 - 0.51 | 0.152 - 0.289 |
|  | Range | 0.165 - 0.302 | 0.187 - 0.279 |  |  |  |
|  | Confidence Interval | 0.220 - 0.247 | 0.216 - 0.243 |  |  |  |
|  | Adjusted P-Value | -- | 0.996 |  |  |  |
|  | P-Value | -- | 0.459 |  |  |  |
| Glutamic Acid | Mean | 1.85 | 1.87 | 1.11 - 2.76 | 0.83 - 3.54 | 1.17 - 2.21 |
|  | Range | 1.29 - 2.33 | 1.45 - 2.48 |  |  |  |
|  | Confidence Interval | 1.67 - 2.03 | 1.69 - 2.05 |  |  |  |
|  | Adjusted P-Value | -- | 0.996 |  |  |  |
|  | P-Value | -- | 0.596 |  |  |  |
| Glycine | Mean | 0.364 | 0.363 | 0.286 - 0.483 | 0.184 - 0.685 | 0.245 - 0.457 |
|  | Range | 0.295 - 0.428 | 0.288 - 0.450 |  |  |  |
|  | Confidence Interval | 0.342 - 0.386 | 0.341 - 0.385 |  |  |  |
|  | Adjusted P-Value | -- | 0.996 |  |  |  |
|  | P-Value | -- | 0.941 |  |  |  |
| Histidine | Mean | 0.297 | 0.291 | 0.191 - 0.380 | 0.14 - 0.46 | 0.202 - 0.345 |
|  | Range | 0.239 - 0.356 | 0.223 - 0.347 |  |  |  |
|  | Confidence Interval | 0.279 - 0.316 | 0.273 - 0.309 |  |  |  |
|  | Adjusted P-Value | -- | 0.996 |  |  |  |
|  | P-Value | -- | 0.321 |  |  |  |
| Isoleucine | Mean | 0.342 | 0.341 | 0.212 - 0.494 | 0.18 - 0.69 | 0.226 - 0.404 |
|  | Range | 0.267 - 0.414 | 0.268 - 0.440 |  |  |  |
|  | Confidence Interval | 0.316 - 0.369 | 0.314 - 0.367 |  |  |  |
|  | Adjusted P-Value | -- | 0.996 |  |  |  |
|  | P-Value | -- | 0.701 |  |  |  |
| Leucine | Mean | 1.22 | 1.21 | 0.687 - 1.83 | 0.60 - 2.49 | 0.695 - 1.51 |
|  | Range | 0.908 - 1.54 | 0.939 - 1.70 |  |  |  |
|  | Confidence Interval | 1.10 - 1.34 | 1.09 - 1.34 |  |  |  |
|  | Adjusted P-Value | -- | 0.996 |  |  |  |
|  | P-Value | -- | 0.709 |  |  |  |
| Lysine | Mean | 0.266 | 0.267 | 0.180 - 0.399 | 0.129 - 0.668 | 0.188 - 0.348 |
|  | Range | 0.210 - 0.333 | 0.218 - 0.330 |  |  |  |
|  | Confidence Interval | 0.242 - 0.291 | 0.243 - 0.292 |  |  |  |
|  | Adjusted P-Value | -- | 0.996 |  |  |  |
|  | P-Value | -- | 0.822 |  |  |  |

**Supplemental Information Table 5** **(continued)**

| **Analyte** | **Reported Statistics** | **Control Maize** | **DP915635 Maize** | **Tolerance Interval** | **Literature Range** | **Reference Data Range** |
| --- | --- | --- | --- | --- | --- | --- |
| Methionine | Mean | 0.214 | 0.200 | 0.106 - 0.315 | 0.10 - 0.47 | 0.151 - 0.255 |
|  | Range | 0.165 - 0.292 | 0.147 - 0.260 |  |  |  |
|  | Confidence Interval | 0.198 - 0.230 | 0.184 - 0.216 |  |  |  |
|  | Adjusted P-Value | -- | 0.301 |  |  |  |
|  | P-Value | -- | **0.0287^*^** |  |  |  |
| Phenylalanine | Mean | 0.496 | 0.489 | 0.302 - 0.735 | 0.24 - 0.93 | 0.299 - 0.612 |
|  | Range | 0.376 - 0.601 | 0.360 - 0.671 |  |  |  |
|  | Confidence Interval | 0.455 - 0.538 | 0.447 - 0.530 |  |  |  |
|  | Adjusted P-Value | -- | 0.996 |  |  |  |
|  | P-Value | -- | 0.488 |  |  |  |
| Proline | Mean | 0.900 | 0.895 | 0.558 - 1.25 | 0.46 - 1.75 | 0.549 - 0.989 |
|  | Range | 0.701 - 1.10 | 0.695 - 1.19 |  |  |  |
|  | Confidence Interval | 0.825 - 0.975 | 0.820 - 0.970 |  |  |  |
|  | Adjusted P-Value | -- | 0.996 |  |  |  |
|  | P-Value | -- | 0.698 |  |  |  |
| Serine | Mean | 0.470 | 0.476 | 0.310 - 0.681 | 0.15 - 0.91 | 0.303 - 0.579 |
|  | Range | 0.320 - 0.593 | 0.370 - 0.592 |  |  |  |
|  | Confidence Interval | 0.432 - 0.509 | 0.437 - 0.514 |  |  |  |
|  | Adjusted P-Value | -- | 0.996 |  |  |  |
|  | P-Value | -- | 0.733 |  |  |  |
| Threonine | Mean | 0.364 | 0.365 | 0.248 - 0.487 | 0.17 - 0.67 | 0.263 - 0.441 |
|  | Range | 0.290 - 0.449 | 0.277 - 0.447 |  |  |  |
|  | Confidence Interval | 0.339 - 0.390 | 0.340 - 0.391 |  |  |  |
|  | Adjusted P-Value | -- | 0.996 |  |  |  |
|  | P-Value | -- | 0.882 |  |  |  |
| Tryptophan | Mean | 0.0572 | 0.0570 | 0.0376 - 0.0990 | 0.027 - 0.215 | 0.0419 - 0.0723 |
|  | Range | 0.0456 - 0.0706 | 0.0436 - 0.0657 |  |  |  |
|  | Confidence Interval | 0.0545 - 0.0598 | 0.0544 - 0.0596 |  |  |  |
|  | Adjusted P-Value | -- | 0.996 |  |  |  |
|  | P-Value | -- | 0.903 |  |  |  |
| Tyrosine | Mean | 0.256 | 0.240 | 0.154 - 0.513 | 0.10 - 0.73 | 0.140 - 0.356 |
|  | Range | 0.128 - 0.357 | 0.135 - 0.322 |  |  |  |
|  | Confidence Interval | 0.235 - 0.277 | 0.219 - 0.261 |  |  |  |
|  | Adjusted P-Value | -- | 0.727 |  |  |  |
|  | P-Value | -- | 0.133 |  |  |  |
| Valine | Mean | 0.454 | 0.453 | 0.306 - 0.629 | 0.21 - 0.86 | 0.329 - 0.519 |
|  | Range | 0.366 - 0.530 | 0.364 - 0.568 |  |  |  |
|  | Confidence Interval | 0.423 - 0.484 | 0.423 - 0.484 |  |  |  |
|  | Adjusted P-Value | -- | 0.996 |  |  |  |
|  | P-Value | -- | 0.893 |  |  |  |

Note: Amino acid unit of measure is % dry weight. Literature range (AFSI, 2019; Codex Alimentarius Commission, 2019; Cong et al., 2015; Lundry et al., 2013; OECD, 2002; Watson, 1982).

^*^ A statistically significant difference (P-Value < 0.05) was observed.

**Supplemental Information Table 6:** Mean and range (minimum and maximum individual values) of minerals in grain from DP915635 maize and non-GM near-isoline control maize (control). Tolerance intervals were derived from Corteva Agriscience’s™ proprietary accumulated data from commercial non-GM maize lines. Literature ranges were obtained from published literature. In-study reference ranges were obtained from the four non-GM commercial maize lines grown at each site.

| **Analyte** | **Reported Statistics** | **Control Maize** | **DP915635 Maize** | **Tolerance Interval** | **Literature Range** | **Reference Data Range** |
| --- | --- | --- | --- | --- | --- | --- |
| Calcium | Mean | 0.00288 | 0.00295 | 0.00144 - 0.00737 | NQ - 0.101 | 0.00178 - 0.00685 |
|  | Range | 0.00175 - 0.00468 | 0.00216 - 0.00479 |  |  |  |
|  | Confidence Interval | 0.00232 - 0.00343 | 0.00240 - 0.00350 |  |  |  |
|  | Adjusted P-Value | -- | 0.996 |  |  |  |
|  | P-Value | -- | 0.433 |  |  |  |
| Copper | Mean | 0.0000912 | 0.0000864 | <0.0000625^a^ - 0.000345 | NQ - 0.0021 | <0.0000625^a^ - 0.000242 |
|  | Range | <0.0000625^a^ - 0.000176 | <0.0000625^a^ - 0.000159 |  |  |  |
|  | Confidence Interval | 0.0000573 - 0.000125 | 0.0000524 - 0.000120 |  |  |  |
|  | Adjusted P-Value | -- | 0.996 |  |  |  |
|  | P-Value | -- | 0.419 |  |  |  |
| Iron | Mean | 0.00146 | 0.00147 | 0.00116 - 0.00332 | 0.0000712 - 0.0191 | 0.00104 - 0.00221 |
|  | Range | 0.00112 - 0.00181 | 0.00114 - 0.00173 |  |  |  |
|  | Confidence Interval | 0.00135 - 0.00157 | 0.00136 - 0.00158 |  |  |  |
|  | Adjusted P-Value | -- | 0.996 |  |  |  |
|  | P-Value | -- | 0.737 |  |  |  |
| Magnesium | Mean | 0.112 | 0.110 | 0.0800 - 0.157 | 0.0035 - 1.000 | 0.0723 - 0.140 |
|  | Range | 0.0901 - 0.139 | 0.0867 - 0.142 |  |  |  |
|  | Confidence Interval | 0.101 - 0.122 | 0.0993 - 0.120 |  |  |  |
|  | Adjusted P-Value | -- | 0.996 |  |  |  |
|  | P-Value | -- | 0.264 |  |  |  |
| Manganese | Mean | 0.000570 | 0.000571 | 0.000325 - 0.00121 | 0.0000312 - 0.0054 | 0.000302 - 0.000991 |
|  | Range | 0.000421 - 0.000756 | 0.000413 - 0.000772 |  |  |  |
|  | Confidence Interval | 0.000513 - 0.000628 | 0.000513 - 0.000628 |  |  |  |
|  | Adjusted P-Value | -- | 0.996 |  |  |  |
|  | P-Value | -- | 0.975 |  |  |  |
| Phosphorus | Mean | 0.318 | 0.313 | 0.211 - 0.413 | 0.010 - 0.750 | 0.201 - 0.374 |
|  | Range | 0.222 - 0.411 | 0.217 - 0.413 |  |  |  |
|  | Confidence Interval | 0.286 - 0.350 | 0.281 - 0.345 |  |  |  |
|  | Adjusted P-Value | -- | 0.996 |  |  |  |
|  | P-Value | -- | 0.412 |  |  |  |
| Potassium | Mean | 0.342 | 0.342 | 0.258 - 0.527 | 0.020 - 0.720 | 0.241 - 0.437 |
|  | Range | 0.273 - 0.444 | 0.298 - 0.448 |  |  |  |
|  | Confidence Interval | 0.319 - 0.366 | 0.319 - 0.365 |  |  |  |
|  | Adjusted P-Value | -- | 0.996 |  |  |  |
|  | P-Value | -- | 0.945 |  |  |  |
| Sodium | Mean | 0.000208 | 0.000232 | <LLOQ^a^ - 0.0141 | NQ - 0.15 | <0.0000625^a^ - 0.00382 |
|  | Range | <0.0000625^a^ - 0.00216 | <0.0000625^a^ - 0.00844 |  |  |  |
|  | Confidence Interval | 0.0000971 - 0.000446 | 0.000108 - 0.000497 |  |  |  |
|  | Adjusted P-Value | -- | 0.996 |  |  |  |
|  | P-Value | -- | 0.825 |  |  |  |
| Zinc | Mean | 0.00183 | 0.00183 | 0.00135 - 0.00343 | 0.0000283 - 0.0043 | 0.00123 - 0.00252 |
|  | Range | 0.00136 - 0.00236 | 0.00158 - 0.00212 |  |  |  |
|  | Confidence Interval | 0.00172 - 0.00195 | 0.00172 - 0.00195 |  |  |  |
|  | Adjusted P-Value | -- | 0.996 |  |  |  |
|  | P-Value | -- | 0.972 |  |  |  |

Note: Mineral unit of measure is % dry weight. Not quantified (NQ); one or more assay values in the published literature references were below the LLOQ and were not quantified. Literature range (AFSI, 2019; Codex Alimentarius Commission, 2019; Cong et al., 2015; Lundry et al., 2013; OECD, 2002; Watson, 1982).

^a^ < LLOQ; one or more sample values were below the assay LLOQ.

**Supplemental Information Table 7:** Mean and range (minimum and maximum individual values) of vitamins in grain from DP915635 maize and non-GM near-isoline control maize (control). Tolerance intervals were derived from Corteva Agriscience’s™ proprietary accumulated data from commercial non-GM maize lines. Literature ranges were obtained from published literature. In-study reference ranges were obtained from the four non-GM commercial maize lines grown at each site.

| **Analyte** | **Reported Statistics** | **Control Maize** | **DP915635 Maize** | **Tolerance Interval** | **Literature Range** | **Reference Data Range** |
| --- | --- | --- | --- | --- | --- | --- |
| β-Carotene | Mean | 0.167 | 0.172 | 0.00197 - 3.68 | 0.3 - 5.4 | <0.0500^a^ - 0.710 |
|  | Range | <0.0500^a^ - 0.340 | <0.0500^a^ - 0.334 |  |  |  |
|  | Confidence Interval | 0.100 - 0.234 | 0.104 - 0.239 |  |  |  |
|  | Adjusted P-Value | -- | 0.996 |  |  |  |
|  | P-Value | -- | 0.728 |  |  |  |
| Vitamin B1 (Thiamine) | Mean | 2.90 | 2.89 | 1.11 - 4.93 | NQ - 40.00 | 1.89 - 3.91 |
|  | Range | 2.18 - 3.68 | 2.40 - 3.57 |  |  |  |
|  | Confidence Interval | 2.64 - 3.15 | 2.63 - 3.15 |  |  |  |
|  | Adjusted P-Value | -- | 0.996 |  |  |  |
|  | P-Value | -- | 0.878 |  |  |  |
| Vitamin B2 (Riboflavin) | Mean | <0.900^a^ | <0.900^a^ | <0.900^a^ - 2.27^b^ | NQ - 7.35 | <0.900^a^ |
|  | Range | <0.900^a^ | <0.900^a^ |  |  |  |
|  | Confidence Interval | NA | NA |  |  |  |
|  | Adjusted P-Value | -- | NA |  |  |  |
|  | P-Value | -- | NA |  |  |  |
| Vitamin B3 (Niacin) | Mean | 14.6 | 14.6 | 7.66 - 31.3 | NQ - 70 | 10.9 - 18.1 |
|  | Range | 12.0 - 19.5 | 12.3 - 20.1 |  |  |  |
|  | Confidence Interval | 12.8 - 16.4 | 12.8 - 16.4 |  |  |  |
|  | Adjusted P-Value | -- | 0.996 |  |  |  |
|  | P-Value | -- | 0.996 |  |  |  |
| Vitamin B5 (Pantothenic Acid) | Mean | 5.68 | 5.67 | 2.49 - 7.52 | 3.01 - 14 | 4.68 - 7.04 |
|  | Range | 4.84 - 7.14 | 4.75 - 6.39 |  |  |  |
|  | Confidence Interval | 5.39 - 5.97 | 5.38 - 5.96 |  |  |  |
|  | Adjusted P-Value | -- | 0.996 |  |  |  |
|  | P-Value | -- | 0.744 |  |  |  |
| Vitamin B6 (Pyridoxine) | Mean | 4.92 | 4.26 | 0.964 - 9.01 | NQ - 12.14 | 1.76 - 10.2 |
|  | Range | 2.39 - 10.7 | 2.84 - 9.36 |  |  |  |
|  | Confidence Interval | 3.93 - 6.15 | 3.41 - 5.33 |  |  |  |
|  | Adjusted P-Value | -- | 0.497 |  |  |  |
|  | P-Value | -- | 0.0771 |  |  |  |
| Vitamin B9 (Folic Acid) | Mean | 1.91 | 1.90 | 0.103 - 2.87 | NQ - 3.50 | 0.290 - 6.98 |
|  | Range | 0.602 - 4.80 | 0.288 - 5.43 |  |  |  |
|  | Confidence Interval | 1.46 - 2.52 | 1.45 - 2.51 |  |  |  |
|  | Adjusted P-Value | -- | 0.996 |  |  |  |
|  | P-Value | -- | 0.975 |  |  |  |
| α-Tocopherol | Mean | 3.67 | 3.97 | 0 - 22.9 | NQ - 68.67 | <0.500^a^ - 25.2 |
|  | Range | <0.500^a^ - 9.83 | <0.500^a^ - 9.97 |  |  |  |
|  | Confidence Interval | 1.48 - 5.87 | 1.78 - 6.17 |  |  |  |
|  | Adjusted P-Value | -- | 0.104 |  |  |  |
|  | P-Value | -- | **0.00147^*^** |  |  |  |
| β-Tocopherol | Mean | <0.500^a^ | <0.500^a^ | <0.500^a^ - 1.10^b^ | NQ - 19.80 | <0.500^a^ - 0.694 |
|  | Range | <0.500^a^ | <0.500^a^ |  |  |  |
|  | Confidence Interval | NA | NA |  |  |  |
|  | Adjusted P-Value | -- | NA |  |  |  |
|  | P-Value | -- | NA |  |  |  |
| γ-Tocopherol | Mean | 21.4 | 22.1 | 0.0611 - 55.4 | NQ - 58.61 | 2.14 - 29.8 |
|  | Range | 3.91 - 33.4 | 2.76 - 29.9 |  |  |  |
|  | Confidence Interval | 15.5 - 27.3 | 16.2 - 28.0 |  |  |  |
|  | Adjusted P-Value | -- | 0.996 |  |  |  |
|  | P-Value | -- | 0.426 |  |  |  |

**Supplemental Information Table 7** **(continued)**

| **Analyte** | **Reported Statistics** | **Control Maize** | **DP915635 Maize** | **Tolerance Interval** | **Literature Range** | **Reference Data Range** |
| --- | --- | --- | --- | --- | --- | --- |
| δ-Tocopherol | Mean | 0.303 | 0.302 | <0.500^a^ - 2.61^b^ | NQ - 14.61 | <0.500^a^ - 0.707 |
|  | Range | <0.500^a^ - 0.881 | <0.500^a^ - 0.687 |  |  |  |
|  | Confidence Interval | NA | NA |  |  |  |
|  | Adjusted P-Value | -- | NA |  |  |  |
|  | P-Value | -- | NA |  |  |  |
| Total Tocopherols | Mean | 25.7 | 26.6 | 0 - 58.8 | NQ - 89.91 | 5.52 - 39.0 |
|  | Range | 4.66 - 39.5 | 3.51 - 36.3 |  |  |  |
|  | Confidence Interval | 18.2 - 33.2 | 19.1 - 34.1 |  |  |  |
|  | Adjusted P-Value | -- | 0.996 |  |  |  |
|  | P-Value | -- | 0.339 |  |  |  |

Note: Vitamin unit of measure is mg/kg dry weight. Not quantified (NQ); one or more assay values in the published literature references were below the LLOQ and were not quantified. Not applicable (NA); mixed model analysis was not performed or confidence interval was not determined. Literature range (AFSI, 2019; Codex Alimentarius Commission, 2019; Cong et al., 2015; Lundry et al., 2013; OECD, 2002; Watson, 1982).

^a^ < LLOQ; one or more sample values were below the assay LLOQ.

^b^ Historical reference data range was provided as tolerance interval was not calculated since the data did not meet the assumptions of any tolerance interval calculation method.

^*^ A statistically significant difference (P-Value < 0.05) was observed.

###

Supplemental Information Table 8: Mean and range (minimum and maximum individual values) of secondary metabolites and anti-nutrients in grain from DP915635 maize and non-GM near-isoline control maize (control). Tolerance intervals were derived from Corteva Agriscience’s™ proprietary accumulated data from commercial non-GM maize lines. Literature ranges were obtained from published literature. In-study reference ranges were obtained from the four non-GM commercial maize lines grown at each site.

| **Analyte** | **Reported Statistics** | **Control Maize** | **DP915635 Maize** | **Tolerance Interval** | **Literature Range** | **Reference Data Range** |
| --- | --- | --- | --- | --- | --- | --- |
| *p*-Coumaric Acid | Mean | 0.0258 | 0.0267 | 0.00773 - 0.0485 | NQ - 0.08 | 0.0126 - 0.0504 |
|  | Range | 0.0181 - 0.0441 | 0.0174 - 0.0457 |  |  |  |
|  | Confidence Interval | 0.0203 - 0.0313 | 0.0212 - 0.0322 |  |  |  |
|  | Adjusted P-Value | -- | 0.497 |  |  |  |
|  | P-Value | -- | 0.0716 |  |  |  |
| Ferulic Acid | Mean | 0.238 | 0.238 | 0.128 - 0.345 | 0.02 - 0.44 | 0.183 - 0.336 |
|  | Range | 0.191 - 0.285 | 0.189 - 0.277 |  |  |  |
|  | Confidence Interval | 0.224 - 0.253 | 0.223 - 0.252 |  |  |  |
|  | Adjusted P-Value | -- | 0.996 |  |  |  |
|  | P-Value | -- | 0.932 |  |  |  |
| Furfural | Mean | <0.000100^a^ | <0.000100^a^ | <0.0000500^a^ | NQ | <0.000100^a^ |
|  | Range | <0.000100^a^ | <0.000100^a^ |  |  |  |
|  | Confidence Interval | NA | NA |  |  |  |
|  | Adjusted P-Value | -- | NA |  |  |  |
|  | P-Value | -- | NA |  |  |  |
| Inositol | Mean | 0.0257 | 0.0267 | 0.00731 - 0.0481 | 0.00613 - 0.257 | 0.0153 - 0.0523 |
|  | Range | 0.0185 - 0.0372 | 0.0180 - 0.0378 |  |  |  |
|  | Confidence Interval | 0.0218 - 0.0296 | 0.0228 - 0.0306 |  |  |  |
|  | Adjusted P-Value | -- | 0.907 |  |  |  |
|  | P-Value | -- | 0.179 |  |  |  |
| Phytic Acid | Mean | 0.970 | 0.972 | 0.504 - 1.33 | NQ - 1.940 | 0.538 - 1.13 |
|  | Range | 0.498 - 1.28 | 0.582 - 1.22 |  |  |  |
|  | Confidence Interval | 0.891 - 1.05 | 0.894 - 1.05 |  |  |  |
|  | Adjusted P-Value | -- | 0.996 |  |  |  |
|  | P-Value | -- | 0.937 |  |  |  |
| Raffinose | Mean | 0.108 | 0.111 | 0 - 0.389 | NQ - 0.466 | <0.0800^a^ - 0.228 |
|  | Range | <0.0800^a^ - 0.216 | <0.0800^a^ - 0.220 |  |  |  |
|  | Confidence Interval | 0.0712 - 0.145 | 0.0744 - 0.148 |  |  |  |
|  | Adjusted P-Value | -- | 0.996 |  |  |  |
|  | P-Value | -- | 0.726 |  |  |  |
| Trypsin Inhibitor | Mean | 2.25 | 2.24 | 1.05 - 8.34 | NQ - 8.42 | 1.25 - 3.82 |
|  | Range | 1.52 - 3.34 | 1.58 - 3.22 |  |  |  |
|  | Confidence Interval | 1.89 - 2.61 | 1.88 - 2.60 |  |  |  |
|  | Adjusted P-Value | -- | 0.996 |  |  |  |
|  | P-Value | -- | 0.908 |  |  |  |

Note: Secondary metabolite and anti-nutrient unit of measure is % dry weight or as indicated. Trypsin inhibitors unit of measure is trypsin inhibitor units per milligram dry weight (TIU/mg DW). Not quantified (NQ); one or more assay values in the published literature references were below the lower limit of quantification (LLOQ) and were not quantified. Literature range (AFSI, 2019; Codex Alimentarius Commission, 2019; Cong et al., 2015; Lundry et al., 2013; OECD, 2002; Watson, 1982). Not applicable (NA); mixed model analysis was not performed or confidence interval was not determined

^a^ < LLOQ, one or more sample values were below the assay LLOQ.

Supplemental Information Table 9: Number of Analytes Below the Lower Limit of Quantification for DP915635 Maize Grain

| **Analyte** | **Number of Samples Below the LLOQ** | | **Statistical Analysis ^a^** |
| --- | --- | --- | --- |
|  | **Control Maize (n=32)** | **DP915635 Maize (n=32)** |  |
| Lauric Acid (C12:0) | 32 | 32 | B |
| Myristic Acid (C14:0) | 32 | 31 | C |
| Heptadecanoic Acid (C17:0) | 1 | 1 | A |
| Heptadecenoic Acid (C17:1) | 32 | 32 | B |
| Eicosadienoic Acid (C20:2) | 32 | 32 | B |
| β-Carotene | 9 | 7 | A |
| Vitamin B2 (Riboflavin) | 32 | 32 | B |
| α-Tocopherol | 4 | 3 | A |
| β-Tocopherol | 32 | 32 | B |
| δ-Tocopherol | 28 | 28 | C |
| Copper | 9 | 11 | A |
| Sodium | 8 | 8 | A |
| Furfural | 32 | 32 | B |
| Raffinose | 12 | 10 | A |

^a^ (A) analyte had < 50% below-LLOQ sample values in both maize lines and was subjected to the mixed model analyses; (B) analytes were not statistically analyzed because values were all below the LLOQ; (C) analytes did not meet criteria for sufficient quantities of observations above the LLOQ and were subjected to Fisher’s exact test
